# Prevalence of Anxiety and Depressive Symptoms and Related Risk Factors among Physicians in China: A Cross-Sectional Study

**DOI:** 10.1371/journal.pone.0103242

**Published:** 2014-07-22

**Authors:** Yanhong Gong, Tieguang Han, Wei Chen, Hassan H. Dib, Guoan Yang, Runsen Zhuang, Yuqi Chen, Xinyue Tong, Xiaoxv Yin, Zuxun Lu

**Affiliations:** 1 Department of Social Medicine and Health Management, School of Public Health, Tongji Medical College, Huazhong University of Science and Technology, Wuhan, Hubei, P. R. China; 2 Shenzhen Health Education and Promotion Center, Shenzhen, Guangdong, P. R. China; 3 Department of Occupational Health, Laiwu Municipal Center for Disease Control and Prevention, Laiwu, Shandong, P. R. China; 4 National Center for AIDS/STD Control and Prevention, Chinese Center for Disease Control and Prevention, Beijing, P. R. China; University of Missouri Kansas City School of Medicine, United States of America

## Abstract

**Background:**

Physicians’ poor mental health not only hinders their professional performance and affects the quality of healthcare provided but also adversely affects patients’ health outcomes. Few studies in China have evaluated the mental health of physicians. The purposes of this study are to quantify Chinese physicians’ anxiety and depressive symptoms as well as evaluate associated risk factors.

**Methods:**

In our study, 2641 physicians working in public hospitals in Shenzhen in southern China were recruited and interviewed by using a structured questionnaire along with validated scales testing anxiety and depressive symptoms. Multivariable logistic regression models were used to identify risk factors for anxiety and depressive symptoms.

**Results:**

An estimated 25.67% of physicians had anxiety symptoms, 28.13% had depressive symptoms, and 19.01% had both anxiety and depressive symptoms. More than 10% of the participants often experienced workplace violence and 63.17% sometimes encountered it. Among our study population, anxiety and depressive symptoms were associated with poor self-reported physical health, frequent workplace violence, lengthy working hours (more than 60 hours a week), frequent night shifts (twice or more per week), and lack of regular physical exercise.

**Conclusions:**

Our study demonstrates that anxiety and depressive symptoms are common among physicians in China, and the doctor-patient relationship issue is particularly stressful. Interventions implemented to minimize workload, improve doctor-patient relationships, and assist physicians in developing healthier lifestyles are essential to combat anxiety and depressive symptoms among physicians, which may improve their professional performance.

## Introduction

Physicians are vulnerable to some mental disorders such as anxiety, depression, and occupational burnout, likely owing to their exposure to high levels of occupational stress [1], [2]. Adverse mental health among physicians can hinder their professional performance and affect the quality of healthcare provided [3]–[5]. These issues will inevitably have negative effects on patients’ health and the development of healthcare system based on manpower sources [6], [7]. Therefore, interest in the psychological well-being of physicians has increased recently, warranting further research regarding factors that influence the mental health of physicians [8].

Several studies have evaluated the mental health of doctors in developed countries such as the US, Britain, Canada, Norway, Japan, and Dutch. Previous studies indicated that the prevalence of depressive symptoms among physicians ranged from 10% to 15% in the US, Britain, Norway, and Japan [9]–[14]. A recent Dutch investigation concluded that anxiety and depressive symptoms were prevalent in 24% and 29% of physicians, respectively [15]. Researchers in China did not examine mental health and psychological well-being among physicians until the beginning of the 21^st^ century. In recent years, a few studies in China focused on anxiety or depressive symptoms among physicians with varying results [16]–[19]. For example, one study showed that the prevalence of depressive symptoms among physicians was 31.7%, while in another study the prevalence of depressive symptoms reached 65.3% [17], [19]. Considering that social, cultural, and economic factors have significant effects on mental health [20], [21], it is necessary to perform additional studies in different regions of a socially and economically diverse country such as China. Furthermore, anxiety and depression symptoms are the two most common mental disorders and commonly occur together [22]. A study that focuses on these two classes of symptoms simultaneously can provide a comprehensive evaluation of their prevalence and identify common potential risk factors. Consequently, we conducted an investigation to quantify Chinese physicians’ anxiety and depressive symptoms as well as evaluate associated risk factors that predispose them to anxiety and depressive symptoms.

## Methods

### Ethics Statement

The study protocol was approved by the Research Ethics Committee in Tongji Medical College, Huazhong University of Science and Technology, Wuhan, China. All the participants read the purpose statement of the investigation and each provided a written informed consent.

### Participants and sampling

This study implemented a cross-sectional survey. Data were collected from June 2009 to October 2009 in the city of Shenzhen-Guangdong Province (Southern China). There were 59 public hospitals in Shenzhen in 2009, and all these hospital enrolled in this study. One quarter of physicians with more than one year of work experience were randomly sampled from the 59 public hospitals. Of 10952 eligible subjects, 2738 physicians were randomly selected, 92 (3.36%) refused to participate, and finally 2646 participants were interviewed through self-administered anonymous questionnaires. Of the 2646 questionnaires distributed, five were discarded because of too many missing data. Finally, 2641 physicians were qualified for this study, with an overall response rate of 96.46%.

### Measurement

The questionnaire includes four sections: socio-demographic information, lifestyles, work-related characteristics, and self-reported health status. Socio-demographic information included gender, age, education level, and marital status. Lifestyle items comprised of sleeping time, physical exercise, and smoking status. Work-related characteristics contained hospital grade, job title, work department, working hours per week, night shift rotation period, and the frequency of doctor-patient conflicts and violence (i.e. “Over the past 12 months, how often did you encounter verbal abuse, threats, intimidation or physical violence by patients and their families?”). Self-reported health status covered two parts: self-perceived physical health and mental health. Self-perceived physical health was measured using a single 5-point Likert scale question ranging from “very bad” to “very good” and coded in the form of values from 1 to 5. Mental health condition comprised of intensified anxiety and depressive symptoms that are measured by the Zung Self-Rating Anxiety Scale (SAS) and the Zung Self-Rating depression Scale (SDS), respectively [23], [24]. Each scale has 20 items presented in a multiple-choice format, and each item is given a severity score from 1 (where very seldom the anxiety/depression symptoms are present) to 4 (where anxiety/depression symptoms are present most of the time). The total score was computed as an original score, and then multiplied by 1.25 to get the standard score with higher scores representing higher intensity of the relevant symptoms. As for the Chinese norm of SAS, a score of more than 50 is considered anxiety symptom. In regards to the Chinese norm of SDS, depressive symptom is defined as a score of 53 or higher [25], [26]. In our research, SAS and SDS have demonstrated high internal consistency; their Cronbach’s alpha coefficient was 0.88 and 0.89, respectively. Additionally their Chinese version has been extensively validated [27], [28].

To reduce information loss, all missing SAS and SDS values were replaced with the calculated mean of a given subject’s complete response to other questions of the respectively scale [29]. For example, if a participant has 2 missing responses in SDS, the values are filled with the calculated average of the remaining completed 18 questions of SDS. However, there were still a few missing items among the categorized variables that were not filled in, such as gender, educational level, and marital status and so on.

### Statistical analysis

All statistical procedures were performed using the Statistical Package for Social Sciences Version 12.0 for Windows. Descriptive analysis was carried out for socio-demographics data, lifestyle items, work-related characteristic variables and self-perceived physical health status. Prevalence of anxiety and depressive symptoms were calculated and compared to socio-demographic characteristics, lifestyles, personal physical health, and work-related characteristics. Chi-square tests were conducted to compare the prevalence of anxiety and depressive symptoms between groups. Multivariable logistic regression analysis was used to analyze the risk factors of anxiety and depressive symptoms (demographic variables, lifestyles, work-related characteristic variables, and self-perceived physical health as the independent variables). Adjusted odds ratios (ORs) and 95% confidence intervals (CIs) for each variable were calculated. For all comparisons, differences were tested using two-tailed tests and *p*-values less than 0.05 were considered statistically significant.

## Results

The study’s participants had a mean age of 39.76 (SD = 9.13) years. More than half of physicians were males (54.71%) and about 70% of physicians had bachelor or post-graduate degree. Almost one third of physicians (35.75%) reported a good self-perceived physical health status, while 13.56% reported poor physical health. Only 20.53% of physicians regularly performed physical exercise, and 30.42% of male physicians were current smokers. With respect to exposure to violence at work, more than 10% of the participants often experienced workplace violence and 63.17% sometimes encountered it (Table 1).

**Table 1:** Descriptive statistics for the characteristics and associations with anxiety and depressive symptoms of the participants.

Variables	Physician		Anxiety symptoms		Depressive symptoms	
	N	%	%	*P*	%	*P*
**Total**	2641	100	25.67	-	28.13	-
**Age, mean (SD** a **)**	39.76 (9.13)		39.26 (8.14)	0.0947^b^	38.67 (8.25)	0.0001^b^
**Gender**				<0.0001		0.0002
Male	1445	57.94	22.01		25.61	
Female	1049	42.06	30.79		32.51	
**Education level**				0.0745		0.0514
High school or less	118	4.48	20.34		23.73	
Vocational School	466	17.68	21.46		24.68	
Bachelor degree	1538	58.35	27.37		30.23	
Master degree	389	14.76	25.19		27.25	
Doctor degree	125	4.74	26.40		22.40	
**Marital status**				0.0998		0.5690
Married/cohabitation	2184	83.01	26.33		27.98	
Single/widow/divorced	447	16.99	22.60		29.31	
**Self-perceived** **Health Status**				<0.0001		<0.0001
Very good	135	5.13	7.41		11.85	
Good	806	30.62	8.93		12.90	
Fair	1334	50.68	26.91		29.84	
Bad/very bad	357	13.56	65.55		62.18	
**Job title**				0.2626		0.0691
Elementary or less	733	28.19	23.60		28.65	
Intermediate	807	31.04	26.15		30.98	
Senior	1060	40.77	26.98		26.13	
**Work department**				<0.0001		<0.0001
Internal medicine	436	16.51	24.31		27.29	
Surgery	441	16.70	26.98		28.57	
Obstetrics and gynecology	322	12.19	35.71		36.96	
Pediatrics	174	6.59	31.03		35.63	
Intensive care	147	5.57	35.37		37.41	
others	1121	42.45	20.70		23.37	
**Hospital grade**				0.0035		0.6288
1	280	10.60	21.07		25.71	
2	1490	56.42	24.30		28.52	
3	871	32.98	29.51		28.24	
**Frequency of conflicts** **and violence**				<0.0001		<0.0001
None	629	23.98	9.54		13.67	
Sometimes	1657	63.17	25.53		28.18	
Often	337	12.85	56.97		54.60	
**Working hours/week**				<0.0001		<0.0001
35–44	788	30.16	16.75		19.16	
45–59	993	38.00	22.76		25.88	
60–69	542	20.74	34.87		36.53	
≥70	290	11.10	42.41		44.14	
**Shift work/week**				<0.0001		<0.0001
0	938	36.17	19.19		20.26	
1	1177	45.39	28.21		32.03	
≥2	478	18.43	32.85		34.73	
**Sleeping times** **(hours)**				<0.0001		<0.0001
≥8	357	13.56	11.20		19.33	
6–8	2126	80.78	26.43		28.08	
<6	149	5.66	49.66		51.01	
**Physical exercise**				<0.0001		<0.0001
Yes	539	20.53	13.73		16.70	
No	2086	79.47	28.72		31.11	
**Smoking**				0.0991		0.0017^c^
Male, No	1002	69.58	20.86	c	23.45	
Male, Yes	438	30.42	24.20		30.59	
Female, No	1021	98.74	30.36		32.13	
Female, Yes	13	1.26	46.15		53.85	

Note: *P* values are associated with Chi-square tests.

a SD is standard deviation.

b *P* value is associated with analysis of variance.

c *P* value is associated with Cochran-Mantel-Haenszel statistics.

The mean standard score of SAS and SDS was 43.09 (SD = 11.10) and 46.08 (SD = 12.04). Overall, there was an estimated of 25.67% physicians with anxiety symptoms, 28.13% with depressive symptoms and 19.01% with both anxiety symptoms and depressive symptoms. Associations between sample characteristics and anxiety or depressive symptoms were assessed using Pearson chi-square tests and the results were displayed in Table 1. Almost all work-related characteristic variables and lifestyle items (except smoking) were associated significantly with anxiety and depressive symptoms. In addition, there were associations between age, gender, work department and anxiety/depressive symptoms.

Table 2 demonstrated the adjusted ORs and 95% CIs for anxiety and depressive symptoms. Compared with male physicians, females physicians had higher odds for anxiety (OR = 1.81, 95% CI: 1.37–2.38) and depressive symptoms (OR = 1.57, 95% CI: 1.21–2.03). Risk factors such as worse self-reported physical health, more violence exposure at work, lack of regular physical exercise, more than sixty working hours per week, and twice or more night shifts per week were associated with anxiety or depressive symptoms. In addition, participants having insufficient sleep were more likely to experience anxiety symptoms than their counterparts. The participants with depressive symptoms were more prone to be current smokers (OR = 1.57, 95% CI: 1.18–2.09).

**Table 2:** Logistic regression analysis of anxiety and depressive symptoms with mutually adjusted odds ratios and 95% confidence intervals.

Variables	Anxiety symptoms	Depressive symptoms
**Age**	1.00 (0.98–1.02)	0.99 (0.98–1.01)
**Gender (Ref** a **: Male)**		
Female	1.81 (1.37–2.38)***	1.57 (1.21–2.03)***
**Education level** **(Ref: Vocational School or below)**		
Bachelor degree	0.92 (0.67–1.27)	1.07 (0.79–1.43)
Post-graduate degree	0.79 (0.52–1.18)	0.84 (0.58–1.23)
**Marital status (Ref: Married)**		
Single/widow/divorced	0.90 (0.65–1.24)	1.17 (0.88–1.57)
**Self-perceived Health Status** **(Ref: Very good)**		
Good	0.90 (0.44–1.86)	1.03 (0.56–1.91)
Fair	2.57 (1.29–5.11)**	2.52 (1.39–4.57)**
Bad/very bad	10.03 (4.87–20.67)***	7.44 (3.95–14.04)***
**Job title (Ref: Elementary or less)**		
Intermediate	0.85 (0.63–1.16)	1.21 (0.91–1.61)
Senior	0.87 (0.57–1.33)	1.06 (0.71–1.57)
**Work department** **(Ref: Internal medicine)**		
Surgery	1.14 (0.77–1.70)	0.98 (0.68–1.42)
Obstetrics and gynecology	1.51 (1.00–2.28)	1.29 (0.88–1.90)
Pediatrics	1.24 (0.77–2.00)	1.26 (0.81–1.97)
Intensive care	1.28 (0.75–2.19)	1.30 (0.79–2.13)
others	1.20 (0.86–1.68)	1.02 (0.75–1.39)
**Hospital grade (Ref: 1)**		
2	1.04 (0.7–1.53)	0.98 (0.69–1.39)
3	1.2 (0.79–1.82)	0.85 (0.58–1.24)
**Frequency of conflicts and violence** **(Ref: None)**		
Sometimes	2.36 (1.67–3.32)***	1.73 (1.29–2.32)***
Often	6.72 (4.38–10.30)***	3.95 (2.69–5.82)***
**Working hours/week** **(Ref: 35–44 hours)**		
45–59	1.04 (0.77–1.40)	1.10 (0.83–1.44)
60–69	1.74 (1.24–2.43)**	1.56 (1.14–2.13)**
≥70	1.94 (1.30–2.90)**	1.90 (1.31–2.77)***
**Shift work/week (Ref: No)**		
1	1.14 (0.85–1.52)	1.27 (0.97–1.66)
≥2	1.52 (1.08–2.14)*	1.40 (1.02–1.93)*
**Sleeping (Ref:** ≥**8 hours)**		
6–8	1.76 (1.17–2.66)**	0.99 (0.71–1.37)
<6	2.70 (1.51–4.83)***	1.58 (0.95–2.64)
**Physical exercise (Ref: Yes)**		
No	1.53 (1.10–2.13)*	1.39 (1.03–1.86)*
**Smoking (Ref: No)**		
Yes	1.18 (0.86–1.62)	1.57 (1.18–2.09)**

a Ref is reference.

*P<0.05;

**P<0.01;

***P<0.0001 (two-tailed test).

## Discussion

### Prevalence of anxiety and depressive symptoms among physicians

Our study demonstrated that anxiety and depression symptoms were common among physicians. Our results confirm previous findings regarding Chinese physicians’ anxiety or depression symptoms. Regarding anxiety symptoms, the mean standard SAS score among our study participants (43.09) was close to that reported by a previous study that applied SAS to assess anxiety symptoms among physicians (46.8 in male physicians and 46.7 in female physicians) [18]. With respect to depressive symptoms, the prevalence among physicians in our study (28.13%) was similar to that reported in a Shanghai-based study among primary-care physicians (31.7%), which used an identical evaluation method [17]. However, the prevalence of depressive symptoms among hospital physicians in the Liaoning Province study was much higher (65.3%) [19]. Our results may differ owing to the participants’ differences in terms of age, gender, and place of residence; additionally this variation might be due to differences in the study design and assessment of anxiety or depressive symptoms. Even though variation existed, in comparison with the Chinese general population, the status of anxiety and depressive symptoms among physicians were significantly higher [30], [31].

The mental health of Chinese physicians participating in our study was poorer than that in some other countries. For example, the prevalence of depressive symptoms among physicians in the US, Britain (intensive care unit physicians), Canada, Norway, Japan, and Benin were 11.3%, 12%, 15.5%, 11%, 8.8%, and 14%, respectively [9]–[14]. The relatively poor mental health status among physicians in China may be attributed to various reasons. The workload of physicians in China continues to increase owing to the aging population, universal health care, and inadequate growth in the number of physicians [32], [33]. Additionally, the doctor-patient relationship in China is perceived as unsatisfactory and medical disputes often occur within healthcare facilities [34]. Finally, physicians’ income in China is lower than that of physicians in many Western countries, and there exists a mismatch between high workload and relative low reward among physicians in China.

### Associated factors of anxiety and depressive symptoms among physicians

The present study indicated that physical health, lifestyles, and work-related conditions are the primary risk factors for anxiety and depressive symptoms among physicians in China. The positive association between physical health and psychological well-being is well documented [35], [36]. Moreover, poor self-perceived physical health status has the largest effect on physicians’ anxiety and depressive symptoms in our fully adjusted statistical model.

With regards to work-related conditions, workplace violence was a significant predictor of physician’s mental health in our fully adjusted model. Physicians who often experienced workplace violence were nearly seven times more likely to be anxious and four times more likely to be depressed compared to those who seldom or never encountered it. Previous studies in Poland and Turkey also demonstrated that workplace violence increases the prevalence of psychological conditions such as anxiety and depression among exposed employees [37], [38]. In our study, more than three-quarters of participants sometimes or often encountered workplace violence. Consequently, the doctor-patient relationship should be improved by the implementation of several measures, including introducing and implementing malpractice insurance for physicians and enhancing doctor-patient communication.

Additionally, our results indicated that physicians who work at least 60 hours per week or who work night shifts twice or more per week were at greater risk of experiencing anxiety and depressive symptoms, which confirms the findings of previous studies demonstrating a significant positive association between lengthy working hours or frequent shift work and anxiety or depressive symptoms [39], [40]. The documented positive associations between very long working hours (more than 60 hours per week) or too frequent night shifts (twice or more a week) within a short period of time and symptoms of anxiety or depression should be carefully considered by hospital administrators or other parties responsible for scheduling physicians.

Lifestyle parameters, such as physical exercise, smoking, and sleeping, were important risk factors for developing anxiety or depression symptoms [36], [41]. Physical exercise serves to restore personal stamina and has been shown to be effective at relieving stress. In addition, there is evidence that regular exercise can improve tolerance towards working shifts, and moderate physical exercise at least three to four hours before sleep is recommended [5], [42], [43]. Approximately one-fifth of physicians in our study exercised regularly, which is lower than the proportion of the general Chinese population that often performs physical activities (28.2%) [44]. On the basis of our results, we suggest that hospitals provide more support in establishing exercise facilities for healthcare workers and encourage them to get involved in exercise activities.

Our study is consistent with a previous study exploring the associations between smoking and symptoms of anxiety or depression [36]. There is evidence that physicians who are current smokers are more likely to experience fewer anxiety symptoms because nicotine can effectively reduce anxiety episodes [45], but there are no data regarding the association between nicotine and depressive symptoms. Additionally, previous studies have indicated that people with sleeping problems were predisposed to psychological disorders such as depression, anxiety disorder, and suicide attempts [46]. In our study, sleeping time was significantly associated with anxiety symptoms, but physicians with fewer than six hours of sleep per day had a tendency to experience depressive symptoms (P = 0.08) [47].

This study has a few limitations that must be acknowledged. First, because this is a cross-sectional study, we cannot establish causality between anxiety or depressive symptoms and their related risk factors. Future longitudinal studies should be conducted to confirm the conclusions from our study. Furthermore, study participants were limited to physicians working in public hospitals in a large city, and the conclusions of this study need to be verified in other areas (e.g., rural areas) and in other types of health facilities (e.g., private clinics) in China. However, our large sample size may increase the validity. Finally, previous studies have shown a moderate correlation between depressive symptoms and occupational burnout [48]–[50], which renders it difficult to distinguish burnout symptoms from those of depressive symptoms and increases the possibility of overestimation of the prevalence of depressive symptoms among physicians.

## Conclusion

We have shown that physicians are at high risk of experiencing anxiety and depressive symptoms. The doctor-patient relationship was a potent source of stress and is strongly associated with physicians’ psychological health. Lengthy working hours and frequent night shifts are also risk factors affecting the psychological health of physicians. In addition, the lifestyles of physicians (including physical activity, smoking, and sleep habits) were shown to significantly affect the risk of anxiety and depressive symptoms. Therefore, interventions implemented to minimize workload, improve doctor-patient relationships, and encourage physicians to develop a healthier lifestyle are essential to combat anxiety and depressive symptoms among physicians, which will improve their professional performance and health outcomes.
